# Use of regulatory cells for achieving functional tolerance of pig heart xenotransplants in humans: a literature review

**DOI:** 10.3389/fimmu.2025.1648926

**Published:** 2025-11-03

**Authors:** Gheorghe Traian Braileanu

**Affiliations:** Department of Surgery, University of Maryland School of Medicine, Baltimore, MD, United States

**Keywords:** pig heart xenotransplantation, regulatory cells therapy, adoptive cell transfer, xenotransplant tolerance, CAR transformed regulatory cells, human recipients, multianalyte biomarkers

## Abstract

Xenotransplantation of pig hearts may help address the current human shortage of human donors once rejection is controlled. One innovative approach to combat rejection in humans is the use of regulatory cell (RC) therapy. The term RC refers to all cell populations that share immunosuppressive functions. The use of RC, including mesenchymal stem cells (MSC) and CD4+CD125lowCD25highFoxp3+ T cells (Treg), may potentially reduce or eliminate the need for chronic general immunosuppression (IS). This approach is hypothesized to act by augmenting suppressive immune mechanisms that maintain tolerance by prevailing over the immune effector mechanisms responsible for rejection. Increasing RC numbers through adoptive cell transfer (ACT) and enhancing their functions via chimeric antigen receptor (CAR) technology are two promising strategies for RC therapy applications. During the various steps of rejection, monitoring specific biomarkers can guide the use of the corresponding RC subpopulation, preferably available off-the-shelf, either alone or in combination, administered once or multiple times. In the future, exosomes or RC-derived active molecules (or their antagonists) may supplement or replace whole-cell therapy. With further research, RC therapy, which has not yet been used in clinics to induce functional tolerance to pig heart xenotransplants in humans, has the potential to become a routine, personalized treatment.

## 1 Introduction

Heart transplantation may be required in patients with advanced heart failure. Currently, more than 6,000 qualified patients for cardiac allotransplantation die each year in the USA due to the shortage of available donors. One possible solution is the use of pigs as potentially life-saving organ donors (1), based on existing anatomical and physiological similarities (2) and general ethical acceptance (3). However, due to antigenic differences between species, a pig heart xenotransplanted into a nonhuman primate (NHP) host without immunosuppressive treatment is likely to be acutely rejected within minutes to hours (4). The use of genetically modified (GM) pigs as donors, together with improved immunosuppressive (IS) treatments of the host, prevented rejection of a pig heart for 9 months in baboons (5) and for 40–60 days in the first two human patients treated at the University of Maryland (1, 6, 7). The development of GM pigs—an important scientific achievement under continuous improvement (8) that contributed to the delay of heart xenotransplant rejection (9)—will not be detailed further, but interested readers are directed to existing reviews (5, 6, 10). IS treatments, by directly reducing the number of available T and/or B effector cells or by blocking costimulatory signals, are a valuable tool to combat acute rejection. However, toxicity, nonspecificity, and side effects (including increased risk for infections, malignancy, or metabolic complications) impede its extended use for chronic rejection treatments (11).

Tolerance without treatment occurs only during normal fetal development as an allograft during gestation (12), in kidney transplants between identical twins (13), or when it appears spontaneously in a minority of liver transplant recipients (14). Novel approaches for induction and maintenance of functional transplant tolerance are being researched and developed.

Currently, continuous antigenic stimulation induced by allo- or xenografts cannot be completely prevented (15). Consequently, the corresponding reactive immune effector responses are continuously amplified. Rejection occurs when the effector mechanisms prevail over inhibitory immune mechanisms at the graft level. Tolerance, in contrast, entails graft persistence despite a progressive increase in effector immune rejection mechanisms. Therefore, a strategy to maintain tolerance should consider the targeted amplification of immunosuppressive mechanisms, specifically, regulatory cell (RC), to act on the immune effector cells during the effector steps of rejection. The specific augmentation of immunosuppressive mechanisms represented by RC, through ACT with one or more populations, to a level prevalent on immune effector mechanisms, could maintain graft tolerance as a possible solution to combat graft rejection without the need for chronic IS.

In this review, the collective term “RC” is used to cover the heterogeneous group of various immune cell populations, each identified by specific morphologic and functional markers, which share immunosuppressive properties. Each RC population, owing to its specific immunoinhibitory effects, has the potential to become instrumental in maintaining tolerance when applied at the corresponding step of rejection. Over the last three decades, an increasing number of RC populations have been discovered, including immune cell precursor populations such as mesenchymal stem cells (MSC) (16) or myeloid-derived suppressor cells (MDSC) (17), as well as immunosuppressors that affect nearly every innate or acquired immune effector mechanism (18–21), which are briefly discussed below.

RC therapy has proven instrumental in clinical use due to its immunosuppressive effects. It is used to treat a wide variety of immune-related diseases, such as GVHD and allergies, or for combating graft rejection in transplantation models (19). Recent successes regarding clinical trials using RC therapy were reported in combating solid organ allograft rejection, especially for the kidney (22, 23). However, for cardiac transplants, only one clinical trial was initiated last year (2024) that uses thymus-derived T regulatory cells (Treg) population to combat allograft rejection in children (NCT04924491). At this point, the use of RC to achieve tolerance to pig heart xenotransplanted in humans has not been studied in NHP models or in the clinical setting. However, based on their mechanisms of action and strong experimental results obtained in different models, RC therapy has the potential to become a routine, effective, and personalized therapeutic tool to combat xenograft rejection. The present review offers arguments in favor of future use of RC as therapy (once or repeatedly, alone or in combination), with emphasis on better studied MSC and Treg, to induce functional tolerance to pig heart xenografts in humans.

## 2 The context of tolerance induction to transplanted grafts

Xenotransplantation activates the existing immune system’s cellular and humoral mechanisms (24, 25) in the background of existing variations due to graft characteristics and host individuality. Host variability (characterized by differences in genetics, age, sex, previous diseases, and antigenic exposure), in addition to early induced postsurgical inflammation and continuous antigenic stimulation generated by the graft, highlights the complexity of immune responses. At the local graft microenvironment, the permanent interactions between inducing factors and reactive effector immune mechanisms can be tentatively grouped into a dynamic succession of general immune steps progressing toward rejection (**Table 1**).

**Table 1:** Tentative enumeration of general immune response steps oriented toward xenograft rejection.

Step		Induced by	Mechanism	Effects	Treatments
1	Sudden inflammation	Surgery	Cellular + humoral	Inflammatory background	Early anti-inflammatory therapy
2	Hyperacute/acute graft rejection	Earlier encounter with antigens similar to the graft ones	Cellular + humoral	Rapid graft damage	Use of IS and genetically engineered pigs successfully delayed acute rejection
3	Activation of innate immune cells (DC, N, M, KC)	Xenoantigens and injury recognition signals	Cellular	Decreased threshold for activation of acquired immune effector cells	Use of DC, N, M, KC regulatory cells
4	Induction of acquired immune effector cell activation	Earlier step	Cellular	Immune effector cell activation. Secretion of cytokines	Costimulation blockade treatments
5	Amplification of acquired immune response	Permanent antigenic stimulation on a dynamic background. Cytokine secretion. Metabolic changes.	Cellular + humoral	Disequilibrium between RC and effector cells in favor of effector cells	Augmentation of RC compartment. Use of cytokines, active molecules
6	Effective phase of acquired immune response	Prevalence of effectors versus RC actions. Cytokine secretion. Metabolic changes	Cellular + humoral	Tissue destruction by direct cellular contact and ADDC	After early detection of rejection by new biomarkers, use specific adoptive RC compartment transfer treatments

Dendritic cells (DC). Neutrophils (N), monocytes (M), killer cells (KC), antibody-dependent directed cytotoxicity (ADDC).

**Figure 1:**
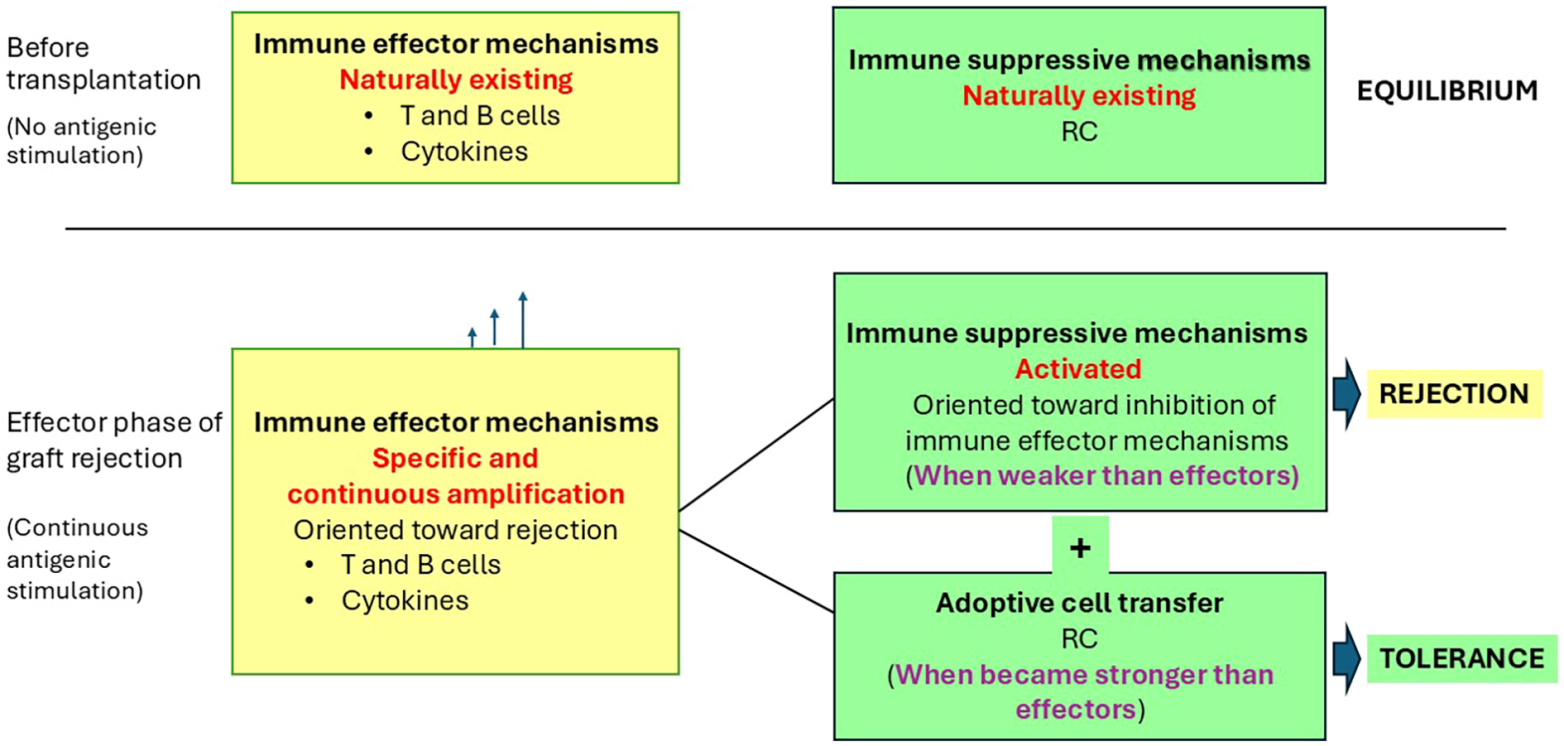
Possible roles of RC in tolerance induction to a transplanted xenograft. Continuous antigenic stimulation due to the presence of the xenograft induces a continuous increase in size or efficiency of the immune effector mechanisms (represented by T and B effector cells and cytokines). When the effects of this increase became stronger than existing local immune suppressive mechanisms (represented by RC—including MSC and Treg), it induced the effective step of graft rejection. ACT of RC therapy increases the size of local immunosuppressive mechanisms. When the cumulative effects of existing and supplemented immunosuppressive mechanisms prevail over the immune effector ones, it may induce tolerance.

Each step (26) may be characterized by the resulting effects of a cascade of immune-reactive mechanisms, each driven by a specific set of cellular subpopulations. Surgery initiates an inflammatory environment (27) (**Table 1**, step 1), which may change the threshold for subsequent innate immune cell activation. Meanwhile, preexisting activated immune cells and antibodies generated by previous encounters with antigens like those of the graft (trained immunity (28)) act immediately, potentially inducing hyperacute or acute graft rejection (**Table 1**, step 2). Activation of innate immune cells (neutrophils, monocytes, dendritic cells, and killer cells) responses (18, 29) is stronger when induced by xenoantigens (30) than when induced by alloantigens (**Table 1**, step 3). Antigen-presenting cells (APC), by direct (31), indirect (32), or semidirect (33, 34) antigen recognition pathway mechanisms, present graft antigens to receptor T lymphocytes. This activation leads to the initiation of cellular and humoral acquired immune mechanisms (**Table 1**, step 4), which, besides antigenic stimulation (signal 1), include costimulation (signal 2) and cytokine secretion (signal 3) (35). Continuous antigenic stimulation induced by the graft (15) leads to continuous amplification of effector mechanisms (**Table 1**, step 5) that, when it prevails over the suppressive action of RC, may induce graft rejection (**Table 1**, step 6) (**Figure 1**). The cellular aspects of the effector immune system are mostly represented by T and B cells. Activated CD4 helper T cells stimulate CD8 cytotoxic effector T cells, which induce cellular-mediated graft rejection through various mechanisms such as specific programmed cell death (36), cell lysis by direct contact (granzymes), and necrosis of graft cells. Rejection also involves the secretion of antibodies by B cells that induce antibody-dependent directed cytotoxicity (ADDC) as the main antibody-mediated rejection mechanism. In NHP, the incidence of antibody-mediated rejection compared to that of acute cellular rejection is much higher after xenotransplantation (46% vs. 7%) than after allotransplantation (3% vs. 63%) (37). Humoral responses, in addition to specific antibody production, also include changes in the cytokine network, which, after interactions with the existing neuroendocrine humoral networks, lead to changes in cellular activation and metabolic modifications (24).

The simultaneous presence of steps 3–5 (**Table 1**) complicates the identification of a specific time or mechanism for targeted prevention of rejection and maintenance of tolerance. Modern molecular techniques can provide data to identify new complex biomarkers that may diagnose and predict graft rejection before visible changes occur. Values above the threshold for one or more specific markers may be tracked during the continuous monitoring of the cumulative effects of complex graft rejection mechanisms and be used as an additional tool to determine when and what treatments or interventions are needed to maintain tolerance. A detected disequilibrium, in addition to ACT therapy with one or more specific RC subpopulation(s) (38–40), may require the use of additional corresponding tools, such as cytokines (41), metabolites, active molecules (specific microRNAs (42, 43) or glycoproteins (44)), or even short-term IS, in order to delay rejection. Therefore, more than one specific intervention may be necessary to achieve tolerance, possibly with one or more RC products ideally available off the shelf.

At any given time point, the net balance between applied therapy and the existing immune cell subpopulations, along with modifications of antibody production, cytokine secretion, or metabolic changes, determines the next step of the immune reactive response. Each effector phase of a reactive immune response may be modulated by immunosuppressive mechanisms of a specific RC subpopulation. When differentiation of effector immune cells and RC is not coordinated (45–47), and the suppressive activity of existing RC (48) is overwhelmed by an increasing number of effector immune cells, rejection may occur. Conversely, stronger immunosuppressive mechanisms of RC over effector immune responses oriented toward rejection may preserve and maintain graft functionality.

In this paper, it is hypothesized that the augmentation of the immunosuppressive mechanisms can induce operational tolerance (**Figure 1**). Prolonged tolerance was obtained by increasing the number, as well as the quality, of RC used as treatment (49) in different allotransplantation clinical settings. ACT with RC, by augmentation of immunosuppressive mechanisms, is proposed to maintain functional tolerance also in human patients who have received pig xenografts (**Figure 1**), especially during the effective rejection step 6 (**Table 1**). Protective mechanisms of RC against heart xenograft rejection are better understood in the context of the complex local cardiac microenvironment and existing inflammation.

### 2.1 Complexity of the heart microenvironment

Immune effector mechanisms of transplanted heart rejection can be better studied and understood at the level of the local cardiac microenvironment (50), besides the secondary lymphoid organs (SLO) (51, 52). The healthy mammalian heart has an estimated two to three billion cardiac myocytes, representing approximately 75% of the normal myocardial tissue volume (50, 53, 54). Other cell types include fibroblasts, resident macrophages, endothelial cells, and perivascular cells (50, 53, 54). Resident immune cells originate from progenitor cells during development and comprise 5% of the cellular population in the human ventricular tissue (50, 55, 56). In healthy mouse cardiac tissue, the number of mononuclear phagocytes, neutrophils, B cells, and T cells is 12-fold higher than that in skeletal muscle, demonstrating the importance of immune cells in maintaining heart homeostasis (50, 57, 58). Immune cells, particularly Treg, also play an important role in various heart pathologies. For example, compared to healthy myocardium, the number of Treg that peaks on day 7 after an infarct, presumably in an antigen-dependent manner, has been shown to be protective (59). In monkeys, the Treg percentage from CD3 cells is elevated in the heart allograft at rejection compared with peripheral blood (PB) (60).

The microenvironment of the heart xenograft reflects various immune-mediated intercellular interactions and epigenetic modifications (46), as well as changes in immune humoral mechanisms of action that influence RC and all constitutive cells. The immune cell secretion of specific antibodies, cytokines, and other soluble mediators (TGF-β, retinoic acid), and their concentrations influence the graft microenvironment and depend on metabolite availability. Immune rejection mechanisms can be further complicated by the simultaneous presence of injury or repair processes, such as dysregulation of fibrosis, which contribute to dynamic changes of local graft microenvironment throughout the lifespan of a transplant (26). The concept of an organized immunological microenvironment, or niche, may help to better characterize the complexity of various rejection steps (61, 62). Focusing on specific compartments, such as adventitial vascular niches, may be useful in understanding and treating rejection.

Further characterization of the differences in cellular composition of the heart between healthy and diseased states (50) by single-cell analysis may identify new biomarkers for rejection and possible new therapeutic targets. Detection of new specific RC subpopulations at the level of the grafted heart during rejection is currently an active field of research (63, 64).

Maintenance of xenograft tolerance may be hypothetically achieved when the immunosuppressive effects of existing local cells are supplemented by ACT of a specific RC population and become more potent than the immune effector mechanisms of rejection (**Figure 1**).

The balanced interactions of various immune mechanisms create a dynamic microenvironment that can be characterized from a functional point of view as proinflammatory or immunosuppressive.

### 2.2 Inflammation and transplant tolerance

The importance of the inflammatory effects on local graft rejection is well established (65). Activated innate immune cells, a major source of proinflammatory cytokines, are considered a barrier to successful pig-to-primate xenotransplantation (66). While the initial inflammatory response and influx of immune cells are essential for limiting and clearing tissue damage, excessive or prolonged inflammation can be detrimental. It may lead to increased cardiac rupture, disproportionate collagen degradation, infarct expansion through phagocytosis of healthy cardiomyocytes, increased left ventricular dilatation (50, 58, 67), and adverse cardiac remodeling (68). Excessive inflammation acts by directly influencing the regulation of effector immune cells, as well as by inducing the production of active cytokines (66).

On the other hand, RC are attracted to the graft and influences rejection mechanisms by suppressing the negative effects of excessive immune cell responses. Prolonged inflammation during an active immune response increases the ability of APC to stimulate effector cell mechanisms, which in turn overcome immunosuppressive controls. RC continuously and proactively (69) suppresses excessive immune responses. A timed increase in the number and functions of specific immunosuppressive RC subpopulations during the different steps of the immune response has the potential to reduce inflammation, which may help maintain xenograft tolerance.

In addition, the interaction between inflammation and coagulation can initiate a cascade of reactions (70), resulting in the uncontrolled production of inflammatory mediators and coagulation factors (30). Theoretically, the systemic inflammatory response precedes, and most likely promotes, activation of coagulation in xenograft recipients (30). The coagulation dysregulation induced by xenografts, leading to thrombotic microangiopathy, may be a cause of graft failure (71). The use of transgenic pigs expressing human coagulation-regulatory proteins (such as tissue factor pathway inhibitor, thrombomodulin, endothelial protein C receptor, or CD39) reduces the dysregulation of coagulation and decreases the amplification of inflammation after xenotransplantation (30), which increases graft survival but does not induce functional tolerance. Other genetic modifications in pigs are currently underway (72). The regulation of inflammation and coagulation in xenograft recipients may be mutually beneficial (30), with possible therapeutic actions on different RC subpopulations oriented toward tolerance induction.

However, continuous antigenic stimulation, accompanied by corresponding cellular and humoral changes, may lead to graft destruction through progressive amplification of effector mechanisms and other consequences of chronic inflammation, such as immune cell exhaustion.

## 3 Use of RC to prevent graft rejection

The idea that the immune system normally includes cell populations with specific inhibitory activity is over 50 years old. Results from day-3 thymectomy experiments in mice published in 1969 (73) led to the discovery of the first population of immune suppressor cells, later identified as CD4+CD127lowCD25highFoxp3+ and named regulatory T cells (Treg) (74). To date, they are the best-studied RC subpopulation. Over the past three decades, other distinct cell populations with immunosuppressive functions have been identified that could potentially be used in transplantation (**Table 2**). In addition to immune cells, precursor populations that have immunosuppressive effects, such as MDSC (17, 75) and mesenchymal stem cells (MSC) (76), may also be used to combat graft rejection. RC related to innate immune responses mechanisms include: a regulatory neutrophil population (RN) (77), regulatory macrophages (RM) (18, 78), tolerogenic dendritic cells (TDC) (18, 79), and regulatory-like NK cells (RLNK) (80). Acquired immune response mechanisms, generated mostly by T and B cells, include actions of corresponding RC populations. Among T regulatory cell (TRC) lymphocytes, besides Treg, other populations identified as RC include CD8+ regulatory T cells (CD8+Treg) (20, 81, 82), CD4+CD8+regulatory T cells (DPTreg) (83), CD4-CD8-regulatory T cells (DNTreg) (84–86), and natural killer T regulatory cells (NKTreg) (87). Other T-cell subpopulations, such as interleukin (IL)-10-secreting T regulatory 1 (Tr1) cells (88), transforming growth factor-beta-secreting T helper 3 cells (Th3) (89), and CD8+CD28− regulatory T cells (90), are adaptively regulatory. Specifically, they acquire regulatory functions following specific antigenic stimulation in a particular cytokine milieu. In addition to TRC lymphocytes, were described B regulatory cells (BRC) lymphocytes (91, 92), represented by different B-specific regulatory cell populations (Breg) that exhibit immunosuppressive regulatory activities (93).

**Table 2:** Abbreviated list of RC populations with their roles in transplantation.

Category	RC population	Evidence level
Immune cell precursors	MDSC	Demonstrated role in transplantation tolerance induction (17)
	MSC	In 2018, 12 clinical trials were registered to study their potential in solid organ allotransplantation settings (114).
Innate immunity	Nreg	Described an intestinal regulatory neutrophil population that reduced acute graft versus host disease for allogeneic hematopoietic cell transplantation in mice (77).
	Mreg	They were administered as immune-conditioning therapy for renal transplant recipients (205).
	DCreg	Their use is proposed for the induction of human kidney or liver graft tolerance (206)
	NKreg	Expanded *in vitro* and proposed as therapy to combat chronic graft versus host disease (80).
Acquired cellular immunity and TRC	CD4+ Treg (Treg)	Successful clinical trials for kidney and liver allotransplantation (173), and one for heart in children (NCT04924491). CAR Treg first clinical trial approved for kidney transplants in 2022 (NCT04817774).
	CD8+Treg	In 2021, they were used in the first human phase I therapy trial in kidney transplant patients (82).
	DPTreg	Identified in the human thymus with roles in the development of single positive TCR (207). The percentage of CD4+CD8+CD127lowCD25highFoxp3+ cells in cynomolgus monkeys’ heart heterotopic allotransplanted graft at rejection was increased compared with PB or LN (60).
	DNTreg	Involved in the long-term survival of rat cardiac xenografts in mice (208). Ameliorate hepatic ischemia-reperfusion injury in mice (209).
Acquired humoral immunity and BRC	Breg populations	CD19+CD5+CD1d high Breg correlated with better kidney graft function (210). CD19+CD24(high)CD38(high)Breg associated with long-term lung graft survival (211). CD19+CD5+CD1dhigh Breg have a protective role in heart transplantation (212).

MDSC, myeloid-derived suppressor cells; MSC, mesenchymal stem cells; Nreg, regulatory neutrophils; Mreg, regulatory macrophages; DCreg, regulatory dendritic cells; NKreg, NK regulatory cells; TRC, T-regulatory cells; Treg, CD4+CD127lowCD25highFoxp3+ cells; CD4+ regulatory cells; DPTreg, double positive (CD4+CD8+) regulatory cells; DNTeg, double negative (CD4−CD8−) regulatory cells; BRC, B regulatory cells; Breg, B-specific regulatory cell populations.

Each of the mentioned RC populations has the potential to contribute to transplant tolerance by specifically suppressing effector immune mechanisms at different steps of the immune responses that led to graft rejection. However, their specific roles in xenotransplantation require further investigation. The diversity of these cell populations may allow for tailored RC therapy to meet the needs of each patient (94). Ongoing efforts to discover and characterize new RC subpopulations with local characteristics (41) may reveal new possibilities for combating rejection (64). For example, based on γδ T-cell mechanisms of action (95, 96), detailed characterization of one or more cell subpopulations with immunosuppressive effects (97) is still pending.

Despite their heterogeneity, some RC populations share common immunosuppressive mechanisms. For example, cell-to-cell contact can induce lysis of target cells through granzyme B secretion, as observed in Treg (98), Tr1 (99), and Breg (100). IL-10-related mechanisms have been demonstrated in MSC (101), MDSC (17), Treg (102), Tr1 (88), and Breg (103). However, the effects of these common mechanisms are distinct (69) because they act on specific cell types present in the local graft microenvironment at particular times.

Moreover, almost every RC population has been shown to be heterogeneous, with distinct subpopulations that differ depending on their origin, mechanisms of action, or localization within specific microenvironments. For example, using the FlowSOM tool for morphologically characterizing human Treg in the blood of patients with systemic lupus erythematosus revealed specific differences among 12 analyzed clusters in terms of their phenotypes and sensitivity to treatment with low doses of IL-2 (41). Likewise, a specific enrichment was reported in two of 14 clusters of CD4+CD8+CD127lowCD25highFoxp3+ cells in cynomolgus monkeys’ heterotopic heart allotransplanted grafts at rejection compared with PB or lymph nodes (LN) (60). The identification of specific RC subpopulations and the characterization of their specific roles for the induction of xenograft tolerance requires more research. Furthermore, single-cell analysis using exhaustive “omics” methods revealed differences at the individual cell level (104). Continuing this analytic approach, it is probable that the use of only active RC-derived molecules may prove effective in maintaining tolerance.

**Figure 2:**
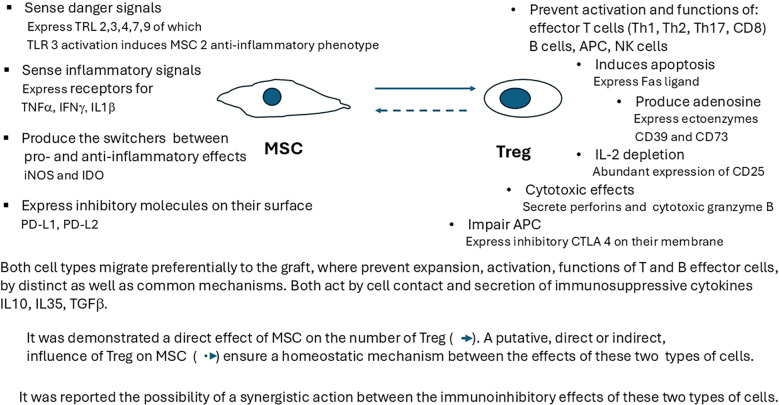
Interactions between MSC and Treg immunosuppressive mechanisms at the xenograft level. Each RC subpopulation has specific mechanisms of action, as well as common inhibitory ones (cell contact, secretion of immunosuppressive cytokines IL-10, IL-35, or TGF-β). It has been demonstrated that the direct effect of MSC on the number of Treg, as well as a synergistic action between the immunoinhibitory effects of these two types of cells, should be taken into consideration when designing specific RC therapies. TRL, toll-like receptor; TNF-α, tumor necrosis factor alpha; IFN-γ, interferon gamma; IL-1β, interleukin 1 beta; iNOS, inducible nitric oxide synthase; IDO, indoleamine 2,3-dioxygenase; PD-L1, programmed cell death ligand 1; PD-L2, programed cell death ligand 2; IL-10, interleukin 10; TGF-β, transforming growth factor beta; CTLA4, cytotoxic T-lymphocyte-associated protein 4.

Every immune effector mechanism appears to have, besides augmentation, a corresponding inhibitory response represented by a cellular population, generally termed as regulatory, that allows for fine-tuning of its mechanisms of action. Consequently, each RC subpopulation, through its specific mechanisms of action, has the potential to combat rejection by acting at a specific step of the immune response (**Figure 2**).

Permanent antigenic stimulation induced by the graft causes an imbalance between the efficacy of continuously amplified immune effector responses and the potency of the inhibitory components of immune responses represented by existing RC (**Figure 1**). When this disequilibrium favors effector responses, graft rejection is induced (**Table 1**, step 6). Detection of an imbalance during continuous blood monitoring after graft transplantation, using specific biomarkers such as cytokines or microRNAs (miRNAs), may signal the need for early intervention. For instance, during step 6 of rejection (**Table 1**), characterized by a severe inflammatory response and tissue damage requiring prompt medical attention, local augmentation of specific RC subpopulations in number (by ACT) as well as qualities (by CAR technology) may maintain tolerance. Further research is needed to improve our understanding of specific immunosuppressive functions of different RC subpopulations (**Table 2**).

### 3.1 ACT of RC and transplantation tolerance

The numeric increase of RC by ACT, particularly for the best-studied MSC and Treg, may be an effective tool for maintaining tolerance in various transplantation settings.

#### 3.1.1 Use of MSC to prevent graft rejection

The possible use of MSC as an approach to maintain tolerance to pig hearts xenotransplanted in humans is supported by the specific properties of these cells and by experimental results. MSC, originally identified in bone marrow (105), are multipotent cells that have the capacity to differentiate into adipocytes, chondrocytes, and osteoblasts (106). Exogenous growth factors added *in vitro* induce the differentiation of MSC into cardiomyocytes, endothelial cells, or smooth muscle cells (107). MSC may function as a cellular reserve, allowing for adaptation to local challenges when needed. The local microenvironment influences MSC via their pathogen-recognition or immune-activation sensing receptors, which generate adaptive changes in their functions. For example, toll-like receptor (TLR)3 activation induces an anti-inflammatory MSC2 phenotype, whereas TLR4 activation induces a proinflammatory MSC1 phenotype (108). The capacity of these cells to promote inflammation when the immune system is underactivated or restrain inflammation when the immune system is overactivated to avoid self-attack (109), is known as their role as “sensor and switcher of the immune system” (110).

MSC infused pretransplant will localize predominantly in lymphoid organs, whereas MSC administered posttransplant migrate preferentially to the graft. Locally, MSC stimulate the proliferation and differentiation of resident progenitor cells and induce immunosuppressive effects by interacting with cells of the immune system. Their suppressive roles prompt their inclusion as RC, especially given their decrease in T and B cells proliferation or activation, increase of T-cell apoptosis (111), induction of a shift in T helper cell balance, and increase in the number of Treg (112, 113) or Breg (103) (**Figure 2**). Inflammatory local graft microenvironments induced by xenotransplantation attract various cell populations, including RC, which begin to influence the existing cellular networks. For instance, MSC, after activating their TLRs that sense damage signals, and their receptors for TNF-α, interferon gamma (IFN-γ), or IL-1β that sense inflammatory status, initiate corresponding adaptive modifications, such as the activation of specific immunosuppressive mechanisms of other existing RC (**Figure 2**). The direct increase of the existing RC pool, induced by activated MSC (**Figure 1**), is hypothesized that in turn influence MSC, forming a putative positive feedback loop (**Figure 2**).

MSC induce the suppression of T lymphocyte proliferation by direct contact and via the secretion of IL-10, tumor growth factor (TGF)-β, hepatocyte growth factor (HGF), and prostaglandin E2 (PGE2) (114) (**Figure 2**). MSC are influenced by, and at the same time influence, different segments of the innate immune response, such as the complement system, toll-like receptor signaling, and various cellular components, including macrophages, dendritic cells, neutrophils, and natural killer cells. For example, MSC can change macrophage phenotypes from a proinflammatory (M1) to a regenerative and anti-inflammatory (M2) state (115). MSC can skew the balance between CD4 effector memory T cells and CD4+Foxp3+ Treg (116) by polarizing both naïve and memory T cells toward a Treg phenotype *in vitro* and by promoting the immune response toward long-term allograft acceptance *in vivo* (112, 117, 118). Interactions between MSC and Treg (119) may help both cell subpopulations survive after ACT (120).

MSC are not recognized by the host immune system because they do not express HLA class II (107) or costimulatory molecules CD80, CD86, or CD40, even after IFN-γ stimulation, making them suitable as an abundant source for off-the-shelf treatment approaches. MSC used for ACT are typically obtained from bone marrow, adipose tissue, gingiva (121), or the umbilical cord blood (122) and are a heterogeneous group of cell subpopulations (108). The present lack of specific markers makes the isolation of a specific subgroup difficult for deeper characterization or treatment (109). However, the use of clonal lines instead of whole-cell populations diminishes variability and enhances the scalability and reproducibility of MSC production and treatments (123). This can be exploited to achieve personalized therapy (124). The quantification of extracellular metabolites (proline, phenylalanine, and pyruvate) in culture and intracellular metabolites (sphingomyelins) can be used as markers to identify MSC lines with high immunomodulatory potency (40).

MSC preferentially migrate toward a graft’s inflammatory environment, where they suppress immune effector responses (114, 125, 126). MSC also have low immunogenicity, are relatively easy to obtain, and are susceptible to transformation by genetic engineering to improve their efficiency (127). Their specific homing mechanisms toward inflammatory environments can also be enhanced *in vitro* (123). The inclusion of bispecific antibodies may be used as a cell-based, specific delivery vehicle for therapeutic molecules (117, 128, 129), such as miRNAs or specific glycoproteins. MSC-derived extracellular vesicles have emerged as promising therapeutic agents for treating cardiovascular diseases (123). Additionally, only MSC-derived exosome miRNAs have demonstrated the potential to modulate immune responses (130) and improve allogeneic heart transplantation outcomes (131). MSC therapy has proven to be an effective tool for controlling graft-versus-host disease (132), treating autoimmune diseases and ischemia reperfusion injury, and mediating wound healing and cardiac repair (133). The immunosuppressive functions of MSC also promote graft survival and, in some instances, have led to functional tolerance (16, 132). Before 2009, there were no clinical reports on their use in transplantation; as of 2018, 12 registered ongoing clinical trials were studying their potential benefits in solid organ allotransplantation settings (114), of which five were for kidney transplants, four for liver, one for lungs, and none for the heart. Preliminary results from kidney allotransplantation demonstrated reduced rejection at 6 months and a partial reduction of tacrolimus doses used for IS (122). Currently, there have been no experiments reported on the use of MSC to induce tolerance to pig heart xenotransplantation in humans, though this is likely to change soon with new innovations.

#### 3.1.2 Use of Treg to prevent graft rejection

Treg make up around 4%–7% of the circulating CD4+ T lymphocytes in healthy mice, NHP, and humans (134), and are proven to be active players in the establishment of peripheral tolerance (135). They prevent low-grade immune activation from becoming an overt immune response if not necessary, suppress ongoing immune responses when no longer needed, limit the negative effects of immune-mediated overreactions induced by chronic inflammation (19), reduce inflammation, and contribute to tissue homeostasis (19, 136). There is a significant correlation between the proportion of Treg and the prognosis and evolution of various solid tumors (137). Specific immunosuppressive effects of Treg have been utilized to prevent rejection of allotransplanted kidneys (138), lungs (139), and livers (140) in humans. In 2022, completed clinical trials investigated the use of Treg to increase tolerance for allotransplanted solid organs—including five for kidney and two for liver—presented positive outcomes and indicated the potential of ACT to induce operational tolerance, although not consistently (141).

Insufficient numbers or compromised functions of RC in the graft may appear due to defects in proliferation (142), increased susceptibility to apoptosis (143), or failure of thymic Treg differentiation or dysregulation (46). Dysfunctional Treg can lead to various immunopathological conditions, including autoimmune diseases, allergies (144), and lethal immunodysregulation polyendocrinopathy enteropathy X-linked syndrome in male subjects (145).

##### 3.1.2.1 Treg mechanisms of action

Treg prevent the activation, expansion, and acquisition of effector functions in a wide range of immune cells, including Th1, Th2, Th17, T follicular helper cells, CD8+ T cells, natural killer cells (146, 147), B cells, and antigen-presenting cells (148), both *in vitro* and *in vivo* (19) (**Figure 2**).

One major action of Treg is the inhibition of autoreactive lymphocytes that escape the thymus or bone marrow checkpoints via contact-dependent and contact-independent mechanisms (149) (**Figure 2**). Their suppressive mechanisms include the production of immunosuppressive cytokines (IL-10, IL-35 (102), TGF-β), the secretion of ectoenzymes CD39 and CD73 that degrade extracellular ATP (a potent pro-inflammatory mediator) to adenosine, which suppresses the immune response (150), and the expression of granzymes or perforins (98) for the destruction of APC (151). *In vitro* assays suggest that Treg do not produce IL-2; however, IL-2 is required by Treg for costimulation and activation (152). The constitutive high expression of CD25 (the alpha chain of the IL-2 receptor) causes these cells to exhibit high-affinity binding to low amounts of IL-2 present during the initiation of an immune reaction; this is a way to suppress the expansion and acquisition of effector functions of conventional T cells (151). Increased constitutive expression of cytotoxic T lymphocyte antigen 4 on the Treg membrane, which binds to the costimulatory ligands CD80 and CD86 on the surface of APC, increases the threshold for T-cell activation. Treg may also act by depleting peptide–major histocompatibility complex (MHC) class II from dendritic cells, which in turn alters costimulation and antigen presentation (153). They also secrete amphiregulin, which supports stem cell proliferation and differentiation (154) by acting on epidermal growth factor receptors (154), and promotes tissue repair (155).

Upon activation under inflammatory conditions, Treg cells express higher levels of effector molecules and become markedly potent suppressors (69). Locally, Treg may enable the establishment of an immunosuppressive environment (102, 156, 157). As previously described, after antigen-specific activation of Treg, some of their immunosuppressive mechanisms can act in a nonspecific mode (i.e., bystander suppression) (102); that is, they may have suppressive effects on neighboring effector cells with different antigen specificities (i.e., dominant suppression) (151). Studies have also demonstrated the survival of Treg beyond the postinfusion time (33): a month for liver transplantation (158), four months in type 1 diabetes patients (159), and one year for kidney transplantation (160). Adoptively transferred Treg may have a lasting effect, even after their disappearance (19), by conferring suppressive capacity to other immune cells located in the graft (102), i.e., infectious tolerance (161), such as Treg with different antigen specificities (162) or Tr1 cells (163). Treg with direct alloantigen specificity are important for tolerance induction, while those with indirect alloantigen specificity are important for long-term tolerance maintenance (151).

Signaling through Foxp3 in mice and humans is essential for Treg suppressive activities (164), although not all cells expressing forkhead box P3 protein (Foxp3) exhibit suppressive activities. This intracellular protein, a transcription factor discovered in 2003, influences the expression of more than 200 genes, including T-cell receptor (TCR)-induced genes. By interacting with over 300 proteins (165), it controls key molecules mediating suppression, influencing differentiation, maintenance, and functional maturation of Treg (19). Studies have demonstrated a complex gene regulation program for Foxp3 that extends beyond the simple model of a transcription factor binding to a gene promoter (46). Specifically, this includes the complex transcription factors RUNX1 and CBFβ, which interact with the demethylated and highly conserved noncoding sequence 2 (166, 167), critical for Foxp3 locus activation during Treg cell maturation (168, 169). SATB1 expression, a pan-histone deacetylase inhibitor that has been shown to increase the acetylation of histones at the regulatory elements of Foxp3, precedes Foxp3 expression in Treg precursors (170) and enhances Treg cell suppressive function, both *in vivo* and *in vitro* (171).

Tolerance induction must also consider the complexity of Treg interactions with corresponding effector cells at different steps of the immune response during graft rejection, as well as the existence of multiple Treg subpopulations (134), each with its specific effects, which act in the local heart graft microenvironment (172). Naturally occurring CD4+Foxp3+ Treg are developmentally determined in the thymus as a distinct cell subpopulation specialized in suppressive functions and form the majority of Foxp3+ Treg in the periphery. In addition, under specific conditions, conventional T cells (T conv) at peripheral sites, such as the intestinal mucosa, can gain stable Foxp3 expression and differentiate into peripherally derived Treg. T conv can also differentiate *in vitro* to express Foxp3 under specific conditions, forming *in vitro*-induced Treg (19). Based on their differentiation status, Treg can be classified as naive, central memory, or effector (173, 174). Naive Treg cells have not yet encountered their cognate antigens and reside in the SLO. After activation by antigen encounters, they differentiate into effector Treg that migrate out of the SLO via circulation into target tissues or become memory Treg (19, 175). The effector Treg are highly proliferative, exhibit strong *in vitro* suppressive activity, and possess a highly demethylated Treg-specific DNA region (CNS2) (19).

Treg have a high capacity to adapt locally to their environment (176) and contribute to tissue homeostasis by controlling inflammation and more specialized mechanisms, such as the production of growth factors (19). For example, there are differences in phenotype, origin, and function between the four well-characterized nonlymphoid Treg populations: visceral adipose tissue Treg (177), intestinal Treg, skin Treg, and skeletal muscle Treg (178). A comparison of Treg taken from the blood, tissue, and tumors demonstrated that while tissue and tumor Treg have greater similarity owing to an activated phenotype, the three groups remain relatively distinct (179). Treg that accumulate in the mouse myocardium after an infarct have a different transcriptome than Treg located in lymphoid organs (172). Additionally, subclinical atherosclerotic plaques are associated with specific Treg subpopulations (180). There are also human Treg subsets with distinct functional and tissue-homing characteristics (41). This demonstrated tissue specificity may suggest that off-the-shelf Treg products that could possibly be designed for organ specific cell therapy (134).

##### 3.1.2.2 Isolation of Treg for ACT

The lack of specific markers for Treg has made their availability for transfer challenging (134). Surface expression of CD25, which correlates with intracellular expression of Foxp3 (39) in mice, NHPs (181), and pigs (182), and is used as a marker for Treg identification from blood, is not highly specific. The use of Foxp3 as a marker is also problematic because it can be transiently expressed in other activated T-effector cell subpopulations. This has led to the identification of additional markers for Treg isolation, such as low CD127 expression, CD49d, CD45RA, LAP (183), GARP (184), Helios (185), Neuropilin 1, CD27 (186), and CD137 (187). The combined use of these markers may increase the purity of Treg suspensions. Single-cell analyses may also lead to future discoveries of new and unique markers for each specific RC subpopulation. Currently, rigorous protocols for isolating highly purified human Treg for clinical applications require further investigation (39).

Due to the low precursor frequency of Treg in circulation, different multiplication attempts have been initiated. Direct *in vivo* use of vitamin D (188), rapamycin (189), and low-dose IL-2 (190) had limited success in transplantation because of the small size of their effects, which are also not specific enough to influence the frequency of only one cell subpopulation. As an alternative, *in vitro* Treg multiplication protocols have been developed (191) for patient cells obtained from peripheral blood (192) and other sources (193). It has also been suggested that CD4+ Treg or CD8+ Treg used for immunotherapy in the future may be derived *in vitro* from embryonic or induced pluripotent stem cells (82).

ACT therapy with Treg can use either the host regulatory cells or cells from another human donor (194), live or deceased, and be utilized *as is* or multiplied (195), and transformed *in vitro*. Currently, Treg for ACT are isolated usually from peripheral blood by leukapheresis through magnetic- or fluorescence-activated cell sorting using a battery of markers, and subsequently expanded *ex vivo*, typically by stimulation with IL-2 and anti-CD3/anti-CD28 coated beads (122) using different GMP protocols (196, 197). The resulting cells, preferably made specific for donor antigens and transformed by CAR technology to improve their functions, can be either administered directly or cryopreserved until near-patient thawing and infusion.

##### 3.1.2.3 Use of Treg in heart transplantation

Considering Treg mechanisms of action and the positive results obtained when used in solid-organ transplantation, it is surprising that ACT with Treg is not in practice to improve heart allotransplantation outcomes in clinics, with only one exception mentioned. The use of Treg in xenotransplantation has been suggested only for the induction (181) and maintenance (39) of tolerance. Reports in the literature support this hypothesis and suggest its application for heart xenotransplants (198). However, the use of Treg to attain tolerance to pig hearts xenotransplanted in humans has not been initiated. Due to ethical considerations, answers to this question must be obtained indirectly, first through NHP experiments. However, the number of tests in monkeys, including baboons, to evaluate this approach is limited. The availability of genetically engineered pigs as potential donors for humans has increased interest in the use of ACT with Treg to induce tolerance to heart xenotransplants (199, 200). The ability of *in vitro* expanded human Treg to inhibit T-cell-mediated rejection of porcine islet xenografts (201) in a humanized mouse model can be regarded as a proof of concept in this direction. The number of Treg in baboon peripheral blood may also be linked with long-term survival of pig heart xenograft (198).

Regarding efficiency, Treg with acquired antigen specificity have proved to be more potent inhibitors than polyclonal Treg (202, 203). Baboon Treg expanded and made specific *in vitro* with pig antigens were 4–10-fold more effective inhibitors of the proliferation of CD4+CD25− baboon T cells than freshly isolated Treg cells (181, 199). Consequently, the use of autologous human Treg exposed *in vitro* to pig antigens may lower the cell count required for effective ACT therapy to induce tolerance to xenotransplanted pig hearts in clinical settings.

Currently, the use of ACT with Treg therapy in human diseases (19) or for inducing tolerance in allotransplanted grafts (33) has been proven safe (141). This supports their application in the context of tolerance induction for xenotransplanted pig hearts.

Given the importance and urgency of addressing pig heart xenograft rejection in humans, and given recent experimental results, safety records, potential elimination of IS, and CAR technology improvements, the use of ACT with Treg is expected to expand in the future (204), potentially making pig heart xenotransplantation a routine procedure in humans.

The application of other RC subpopulations to enhance heart xenograft tolerance through ACT at specific rejection steps in humans requires further development and characterization.

#### 3.1.3 Use of other RC populations in transplantation

Targeting ACT toward one specific immune cell effector population (starting with cells that build innate immunity and ending with effector cells of acquired immune responses) has demonstrated the potential of various RC populations to become important tools for the induction of graft tolerance. However, with few exceptions other than MSC and Treg, such treatments are generally at the initial stages of clinical development (**Table 2**).

MDSC are a group of heterogeneous precursor immune cells differentiated from hematopoietic stem cells in the bone marrow that mainly inhibit T-cell proliferation and activity, as well as promote angiogenesis (75). In peripheral immune organs, MDSCs become dendritic cells, granulocytes, and macrophages. Their immunosuppressive functions are mediated via multiple pathways, such as arginase-1, nitric oxide synthase, reactive oxygen species, indoleamine-2,3-dioxygenase, heme oxygenase-1, prostaglandin E2, cyclooxygenase-2, or cytokine secretion, such as IL-10 and TGF-β (17). At the local organ microenvironment, MDSCs are part of the immunosuppressive network, interact with other immune cells, and may generate positive feedback loops. For instance, their survival and proliferation have been proven to be compatible and mutually complementary with Treg (213), potentially increasing their common effects on T-cell suppression. ACT with MDSC expanded *in vitro* has also been proven to be useful to maintain allograft tolerance (17, 214).

As innate immune RC populations, regulatory macrophages (Mreg) are a subtype of macrophages that are involved in regulating the immune response by inhibiting activated T lymphocyte proliferation (215). After the administration of Mreg to two renal transplant recipients, the authors suggested this approach as immune-conditioning therapy for solid organ transplantation that requires further study (205). Regulatory dendritic cells (DCreg) may induce the differentiation and expansion of Treg and secrete immunomodulatory cytokines, such as IL-10 and TGF-β (216). The manipulation of DCreg was proposed for the induction of human renal and liver transplantation tolerance (206). In other studies, regulatory natural killer cells were expanded *in vitro* and proposed as a clinical therapy to combat chronic graft-versus-host disease (80). Neutrophils have been described as central regulators across all stages of tumor evolution (217) and the different steps of graft rejection. In recent years, a growing number of neutrophil subpopulations have been described (218), some of which may have regulatory functions. For example, an intestinal regulatory neutrophil population that reduced acute graft-versus-host disease in allogeneic hematopoietic cell transplantation has been identified in mice (77).

TRC subpopulations suppress various effector mechanisms of acquired immune responses (39). CD8+Treg are considered true Treg with cytotoxic function (20). In a xenotransplantation pig-to-rat corneal model, xenograft survival was prolonged by the adoptive transfer of T regulatory CD8+CD28− cells (219). The role of CD8+ Treg (20) has led to their use in the first human phase I therapy trial in kidney transplant patients in 2021 (82). Donor double-negative Foxp3+ Treg that promote allogeneic mixed chimerism and tolerance (220) have been proposed as a potential tool in xenotransplantation (39). Double-positive (DP) (CD4+CD8+) cells have been associated with graft rejection in a nonhuman primate model of islet transplantation (83). Their numbers also increased in the blood of liver transplanted patients (221). In the human thymus, a DP population with regulatory properties, with assumed roles in single positive regulatory cells development, has been described (207). Single-cell RNA sequencing in a cynomolgus monkey model has demonstrated the heterogeneity of DP cells, some of which exhibit a phenotype consistent with regulatory functions (222).

Tr1 cells are classified as a distinct subset of T cells that lack constitutive Foxp3 expression and exert suppressive functions primarily via the secretion of IL-10 and TGF-β. They are susceptible to CAR technologies after multiplication *in vitro* and maintain a stable phenotype in inflammatory environments. Findings also suggest possible heterogeneity among Tr1 cells. Their capacity to suppress immune responses against specific antigens was confirmed in mouse and rhesus monkey models of pancreatic islet transplantation (88) and was used in two clinical trials for kidney transplantation (223).

B cells are major players in antibody-mediated graft rejection mechanisms through the production of specific antibodies by plasma cells, known as B effector cells (224). B cells are also known as secondary antigen-presenting cells and as sources of immunoregulatory cytokines (91). Consequently, immunosuppressive Breg appear as an important immunomodulatory tool. By suppressing inflammation and/or antibody production, they may induce positive effects on mechanisms of tolerance induction in xenotransplants (225). Their immunosuppressive effects are induced through the secretion of soluble molecules, such as cytokines (IL-10, IL-35, or TGF-β) (226) and cytotoxic enzymes (granzyme B), or by direct cellular contact through the molecules expressed on their surface (MHC II, costimulatory CD80, CD86, CD40, or ligands for Fas or PD-1) (100). Studies on chronic inflammatory responses in patients with systemic lupus erythematosus, other autoimmune diseases, and allergies (227) documented a lack or functional deficit of circulating Breg. In various animal models, Breg have been shown to suppress autoimmune responses in experimental autoimmune encephalomyelitis (228), collagen-induced arthritis (229), and spontaneous colitis (230). In humans, Breg are also involved in multiple sclerosis (231), atopic dermatitis (232), allergic diseases (233), and kidney transplantation (234). Breg are, in fact, composed of a pool of different B-cell subpopulations with rather heterogeneous phenotypic and transcriptional properties (176). For instance, there is a difference in the dynamics of three subclasses of Breg cells during acute cellular rejection and chronic allograft dysfunction after lung transplantation (47). Assessment of Breg/Beffector balance may identify patients who require more immunosuppression (225). The role of Breg in the induction of immune tolerance to solid-organ transplantation is not completely understood (235) but was presented as a possible key regulator (93, 235–237). The use of B regulatory cells as a tool to combat heart pig xenograft rejection in humans was only proposed last year (37). So far, no therapeutic approach uses the intentional modulation of the frequency or activity of Breg in clinical heart xenotransplantation settings (227), but this is expected to change in the near future.

### 3.2 Use of CAR-transformed RC to improve long-term graft survival

Augmentation of RC immunosuppressive effects can be achieved by increasing their number and by enhancing their qualities. CAR technology has revolutionized the field of targeted cellular therapy (238). RCs are susceptible to molecular transformation to achieve desired characteristics by using CAR technology, an approach studied notably using Treg (134). CAR is a synthetic protein that consists of the fusion of: an extracellular domain that is specific for antigen recognition, a hinge region that provides molecular flexibility, a transmembrane domain that anchors the receptor in the plasmalemma, and an intracellular signaling domain (specifically, CD3zeta, a component of the T-cell receptor) (239). Five generations of CAR have been developed with continuous structure optimization of mainly the intracellular domain (240). The Treg transformed *in vitro* with CAR technology are more efficient than polyclonal Treg for ACT (241). An advantage of CAR-transformed cells is that they bind to antigen in an MHC-independent manner, increasing the number of patients that could be treated with the same batch of cells ready for an off-the-shelf approach (102). The pig heart xenotransplant model offers a major advantage: it allows for the targeted delivery of CAR-transformed RC to the graft because of its unique antigenic properties. Engineering a chimeric receptor that recognizes a swine histocompatibility antigen could potentially direct RC specifically to the heart xenograft. Alternatively, targeting a pig-specific molecule found in the vascular niche could provide even more precise localization. A second transformation may offer the possibility to create a specific carrier of useful molecules (such as a specific cytokine, miRNA, glycoprotein, or a tissue repair molecule) to be delivered at the local graft microenvironment to complement RC suppressive activity. Another important advantage of CAR technology is its potential to achieve targeted immunosuppression that may not interfere with the recipient’s general immune system response, unlike IS (242).

CAR-transformed cells were recently improved by the use of other procedures other than viral vectors for transformation, such as clustered regularly interspaced short palindromic repeats (CRISPR)/CRISPR-associated protein 9 (Cas9) technology (243). There are ongoing efforts to design a “super” Treg (244) by genetically engineering it (245) to increase RC qualities, such as viability, stability, efficiency, or trafficking (134, 203). The use of such specific cells as a “living drug” (246), due to their improved qualities, is expected to reduce the number of RC needed at the level of the graft to prevent rejection. Also in use are Treg designed for Foxp3 overexpression (247, 248), enhanced IL-2, IL-10, IL-35, or granzyme B secretion (249), and antigen-specific receptors overexpression (203).

Currently, multiple editing events are being studied to assess if they can better modulate RC functions. Strategies include the development of dual antigen-activating systems, induction of bioactive protein switches (such as AND or NO) (250), and the addition of a suicide cassette to prevent or block potential adverse events. CAR RC therapy may benefit from the tests of new mRNA-based CAR T therapies that induce cellular changes *in vivo*, avoiding the costs of *in vitro* modifications (251), or from the use of intracellular synthetic circuits proven to regulate protein secretion in human cells (252) by enabling control over local microenvironments. Finally, a drug-induced regulation of engineered cytokines (253) was presented as proof of concept that CAR-T-cell proliferation and activation could be turned on and off at a precise location, in a time-dependent manner via drug administration (254).

CAR-transformed T-cell therapies that have been used successfully to treat hematological malignancies (255) are on course to become potential treatments against solid tumors (256) and are being tested in a large number of clinical studies for other practical applications beyond cancer. Tailoring RC properties with CAR constructs to increase tolerance to organ transplantation is an active field of research (257–260). Applying this strategy to the different RC populations may be helpful in transplantation. For example, CAR-transformed regulatory NK cells may possess greater off-the-shelf potential (240, 261), and regulatory CAR-macrophage cells may have superior tissue infiltration capabilities, enabling them to more effectively penetrate dense tissues (240, 262). Recommended by specific biomarkers, ACT with a small number of corresponding CAR-transformed RC has the potential to balance a specific, detected disequilibrium. However, CAR-modified RC therapy to induce tolerance to heart pig xenograft in humans has yet to be initiated.

### 3.3 The ACT of RC may be replaced with its active molecules

Building on the positive results obtained with cell-based therapies, the use of immune cell-derived exosomes was proposed as an effective substitute for whole cells (263). This approach, compared with cell therapy, is relatively easier to apply and is inexpensive. Exosomes were shown to improve cardiac tissue repair after myocardial damage (264) and enhance the immunotolerance of cardiac allografts (131). However, the use of RC-derived exosomes for heart xenotransplantation requires further investigation (265).

The use of only one specific active molecule (266) for functional tolerance may be a viable alternative. The pig xenotransplanted heart in cynomolgus monkeys exhibited a total of 3784 differentially expressed genes compared to the non-transplanted heart transcriptome, of which 2443 were upregulated and 1305 were downregulated (24). Their analysis, together with single-cell RNA technology, may identify new tolerance-inducing molecules based on the immune mechanisms at each step of rejection, which can be applied to future clinical trials. For example, the activation of inflammasome-related components, including caspase-1 expression, is seen in allografts during the first 7-day posttransplantation. Administration of a caspase-1 inhibitor, VX765, during this interval has been shown to improve graft heart survival and function (267).

The dynamics of cytokine secretion in the local microenvironment, influenced by the immune response or IS treatments, change RC activities, which, in turn, change the cytokine balance (268). This chain of reactions can be targeted for tolerance-induction therapies. For example, IL-35 stabilizes the Treg phenotype to protect cardiac allografts in mice (269), IL-7 improves the fitness of regulatory T cells for adoptive transfer (270), and IL-10 or IL-27 exhibits anti-inflammatory activity (271).

miRNAs, as small noncoding RNA molecules, can be used as promising biomarkers (42) for the detection and prognosis of humoral as well as cellular heart graft rejection in humans (272). They may assess the efficiency of antirejection treatments and may also be used as therapy to induce or maintain tolerance (42). Analysis of their effects on immune responses may lead to the discovery of new mechanisms of rejection (273). For example, miRNAs can directly or indirectly down- or upregulate Foxp3 expression. miR-155 is a promising theranostic agent as its levels are upregulated during both acute rejection and vasculopathy development (274, 275). Specific RC-derived miRNAs or their antagonists could potentially be used in certain circumstances instead of whole-cell therapy. Different RC-derived glycoproteins (44) may have similar potentials.

Recent findings have suggested that metabolite availability is a fundamental determinant of adaptive immune responses (276). The metabolic influences, which regulate the switch between Th17 cells and Treg, include changes in several pathways, such as fatty acid and lipid synthesis (277), polyamine metabolism (278), glycolysis, and ROS control (279). Using differentially expressed gene analysis (570 million raw RNA sequence reads), 21 key biological pathways involved in the terminal stage of pig heart tissue graft rejection on day nine in cynomolgus monkeys were detected (24). Among these that represent cellular interactions in porcine rejected xenotransplanted hearts, some were downregulated, such as arrhythmogenic right ventricular cardiomyopathy, calcium signaling pathways, natural killer cell-mediated cytotoxicity, and the renin–angiotensin system, and some were upregulated, such as pyrimidine metabolism and the p53 signaling pathway (24). Extracellular matrix receptor interactions are involved in signaling events that regulate cell survival, growth, shape, differentiation, migration, or motility (280), as well as in heart remodeling (24, 281). Furthermore, the metabolites produced by commensal bacteria promote the generation of peripheral Treg (282). New therapeutic interventions at the metabolic level may improve clinical outcomes of rejection at the molecular level. In addition, harnessing the nutrient-mediated influence, described as signal 4 in T-cell immunity (35), as a possible adjuvant strategy for the treatment of different diseases (283) has the potential to be used for tolerance induction too. Small-molecule inducible gene regulatory systems in mammalian cells may be an important tool in the future for precise regulation of biological systems (284).

### 3.4 Potential biomarkers for the use of personalized therapy with RC

From the multitude of existing possibilities, the use of a certain RC subpopulation might be directed by specific biomarkers.

Ideal biomarkers should detect rejection before the appearance of morphological modifications of the graft, signaling the need for the use of a specific RC subpopulation to correct a defined, detected disequilibrium of the effector immune response or to track treatment. They should also be easy to operate and manufacture. Such biomarkers for heart pig xenograft in humans still need to be discovered and validated.

The proteins currently used for the evaluation of heart health have limitations. Troponins detect myocardial injury only after necrosis, creatine kinase is less specific than troponins, natriuretic brain peptide is influenced by age or kidney function, and C-reactive protein is a marker of inflammation with low specificity. Recently, glycoproteins have been considered as an additional possible biomarker (44). A gene expression panel to detect rejection, approved by the FDA, requires specialized personnel, is expensive, and may give uncertain results in unique circumstances. The detection of circulating nucleic acids offers possibilities for new biomarkers that are easy to detect in liquid biopsies (285). From these, donor-derived cell-free DNA effectively identifies acute cellular rejection and antibody-mediated rejection, yet cannot distinguish between the two (286) and is not useful for diagnosing patients who have had numerous transplants (263). miRNAs are emerging as the most promising future biomarkers that, by suppressing the expression of specific mRNAs, offer many possibilities for precision medicine. A multitude of miRNAs have been proposed as biomarkers for cardiac development and pathology. Of more than 1,817 human miRNAs associated with various diseases, 150 were identified as playing a critical role in cardiovascular system physiology. For instance, miR-1, miR-133a, miR-208, and miR-499 were identified as the most abundant miRNAs expressed in myocardial tissue, involved in the regulation of cardiomyocyte differentiation in the early stages of heart development (285). In heart allotransplant patients, miR-139-5p, miR-151a-5p, and miR-186-5p demonstrated increased expression at rejection (287). Circulating miR-182a-5p was also identified as a potential biomarker of acute cellular rejection in heart transplantation (288). In the serum of alloheart transplanted patients, 12 miRNAs that accurately discriminate acute cellular rejection, and 17 miRNAs for antibody-mediated rejection, were identified and proposed to be used together under a provisional patent (272). New miRNA biomarkers are continuously being discovered that, after validation, may be used for developing targeted personalized therapy.

The best diagnostic and prognostic data of heart graft rejection in the future may be from multianalyte biomarkers. This means a specific panel of complementary biomarkers that integrate data from multiple assays (such as genomics, transcriptomics, proteomics, specific miRNAs, epigenetic modifications, or extent of nucleosome fragmentation), all obtained simultaneously from the same liquid biopsy sample. All this may be possible to obtain due to modern technical advances, such as new-generation sequencing, digital PCR, and high-throughput data analysis tools. Integrated molecular signatures (263, 289) presumptively answer a question better than each individual component. The use of confirmed miRNAs for the diagnosis or prognosis of heart graft rejection has not been validated for large clinical use, although with further research, this might change.

When expression of specific biomarkers (for example, miRNAs) for an immune effector mechanism (such as excessive inflammation) in the recipient’s serum is matched with the therapeutic effects of a unique RC population, selected from the multitude of existing possibilities based on mechanisms of action, effective precision medicine therapy may be applied.

### 3.5 Challenges and future research directions

Future research experiments are necessary to advance granular knowledge regarding the steps of immune rejection mechanisms to effectively apply RC therapy. Additionally, in-depth characterization and validation of the specific effects of different RC subpopulations in clinical trials are also needed. For example, future studies may focus on identifying unique immune inhibitory effects of regulatory mesenchymal stem cell subpopulations to determine which subpopulation, and when, should be used in the prevention of rejection. In addition, validation of specific and combined biomarkers is essential for the targeted use of RC subpopulations and for monitoring their effects.

As with any other type of cell therapy (241), mechanistic studies regarding RC-based interventions face challenges in terms of logistics (i.e., timing, dosage, frequency) and design of good manufacturing practices for their production and standardization (290). For example, RC administration should consider the circadian rhythms of leukocyte activity, as demonstrated in the literature for CD8+ T cells (291). Furthermore, multiple infusions of *ex vivo*-expanded polyclonal Treg within the first few weeks after transplantation result in inferior graft function (292), most likely due to the early posttransplantation inflammatory environment. Existing clinical trials with RC test different doses (between 10^5^ and 10^7^ cells/kg) and different frequencies of administration to establish the best-use guidelines. The biological variability of RC subpopulations may represent another challenge; the use of clones may ensure better result consistency. More research is also needed for a better understanding of unforeseen off-target immunosuppressive activities (33).

An important question that must be fully addressed before clinical application of RC therapy is its safety profile (240). In rare cases, the ACT of CAR T cells directed against tumor antigens resulted in adverse effects such as cytokine storms and cytotoxicity (293). Further study of T-cell exhaustion induced by tonic signaling of CAR (294) may be important to ensure consistent results of the proposed cell therapy. The *in vivo* stability of transferred RC subpopulations, especially the CAR-transformed ones, also requires more research (295). It is important to overcome the difficulty in establishing the persistence of infused therapy cells in the patient. The use of deuterium, which proved useful in tracking Treg in patient blood or grafted kidney (NCT02088931), was also suggested to track CD8+ Treg (82). An additional challenge is establishing how the local inflammatory background (30, 66, 296) may impacts the phenotype and functions of infused Treg (297). Finally, specifically in the case of using GM pigs as heart donors for humans, the possibility of reactivation of porcine endogenous retroviruses (298) or cytomegalovirus (7) within the xenograft must be addressed (3).

Special attention should be given to the availability and production of necessary therapeutic cells. As for autologous therapy, not all patients are able to donate a sufficient volume of blood to obtain the necessary cells, or intrinsic Treg dysfunction may be present in some patients. The preparation of products ready for an off-the-shelf approach must use a GMP protocol. These protocols should provide solutions to address the timeframe (several weeks in culture) needed to obtain a sufficient number of cells, as well as ensure the purity and sterility necessary for clinical administration, and reduce the high cost of production driven by the need for specific facilities and skilled personnel (173).

## 4 Discussion

Specific and dynamic therapy using the appropriate RC subpopulations is hypothetically an effective approach to maintain graft tolerance. Continuous antigenic stimulation generated by the graft, in an inflammatory background, induces continuous amplification of local reactive effector immune mechanisms. Rejection is hypothesized to occur when these harmful immune effector mechanisms outweigh the immunosuppressive effects of existing RC (**Figure 1**). Consequently, detecting such a disequilibrium through a set of specific biomarkers should trigger a timely intervention with the corresponding RC subpopulation to prevent the chain of immune reactions from progressing toward graft destruction (**Table 1**). This viewpoint highlights the possibility that continuous posttreatment monitoring, in addition to assessing the effects of the initial intervention, may detect new, specific disequilibria in the patient over time. This may require a new specific intervention with the same or another RC subpopulation or other corresponding approaches. The repetition of this pattern, as necessary, may ensure tolerance maintenance based on precision therapy.

The hypothesis that enhancement of existing local immunosuppressive mechanisms, when needed to overcome the immune effector mechanisms of rejection by ACT using RC therapy, may help maintain graft tolerance (**Figure 1**) is supported by theoretical considerations, existing experimental results, and ongoing clinical trials.

ACT using specific RC subpopulations therapy has multiple advantages. It targets specific immune effector mechanisms, in contrast to IS, which nonspecifically dampens the entire immune response. By amplifying existing immune suppressive mechanisms, RC therapy has the potential to be safe and effective within defined parameters, as demonstrated by current evidence. The considerable number of RC subpopulations enables targeted interventions at each stage of rejection, tailored to individual needs. The low immunogenicity of several RC subpopulations makes them suitable for application to many recipients and is ready for an off-the-shelf approach.

RC therapy by ACT is continuously improving. Early attempts using polyclonal RC populations, which had the potential to induce larger-than-intended suppressive effects, have been replaced with the use of antigen-specific populations instructed *in vitro*. The advent of CAR technology has further increased the specificity and efficacy of RC therapy. The initial use of viral vectors to improve RC qualities had the potential of oncogenesis due to the possible random insertion of added genetic material near oncogenes, and has since been replaced with the introduction of CRISPR/Cas9 technologies. The potential apparition of unforeseen effects can be avoided by the insertion of a suicide cassette that can be activated as needed. Using more than one modification provides focused approaches to further improve RC qualities. However, these challenging refinements imply higher costs, specialized personnel, and expensive equipment, although continuous improvements are being made. The main challenge before any clinical application is to validate the safety of RC therapy.

There are other challenges related to standardization and quality control in cell production, such as cell source (which can potentially be mitigated with the use of stem cells), variability (which can potentially be mitigated with the use of cell lines), and ensuring sterility. There is a need for uniform good laboratory practice (GLP) protocols along with clarifications of ethical and regulatory issues (299).

Ideally, an effective RC treatment should maintain functional tolerance by transferring a reduced number of cells with improved qualities, predictable, specific, and long-lasting effects, as well as be cheap and easy to manufacture following GLP protocols. Existing RC therapies have proved helpful in increasing graft tolerance in different transplant settings.

RC therapy will continue to move forward for the induction of functional tolerance with an improved understanding of the different steps of graft immune effector rejection mechanisms (300), detailed characterization of the existing RC populations’ specific roles in suppression, as well as discovery of new RC subpopulations (69) and new specific biomarkers. This knowledge will allow targeted interventions for specific situations as an effective and personalized strategy. As the borders between the rejection steps (**Table 1**) are not well defined, the first challenge for RC cellular therapy to induce tolerance consists of accurate identification of the critical disequilibrium that needs correction. This challenge could be solved by the discovery of new biomarkers. Specific molecules such as cytokines, glycoproteins (44), one or more donor-derived cell-free DNAs (301), or miRNAs (302) are the best candidates to build a battery of multianalyte biomarkers. Further research is needed to analyze which combinations of signals need to be monitored to better characterize a specific immune effector mechanism (69). The combinatorial multibiomarker approach offers higher sensitivity than each component alone, ensuring the highest area under the curve (303) for the detection of a specific endpoint important for tolerance treatment. The next challenge is to choose the appropriate RC subpopulation or product for ACT, based on its specific mechanism of action. Continuous monitoring of serum levels (liquid biopsies) may indicate when and what interventions are required, based on individual threshold values. Consequently, maintenance of functional tolerance is a dynamic process that involves suppression of various immune effector mechanisms at different steps of rejection and may require multiple interventions. These could include using various cell subpopulations or their off-the-shelf products as indicated by specific biomarkers. The enhancement of immunosuppressive effects of a specific RC subpopulation can be achieved by increasing cell numbers via ACT, as well as by improving their qualities via CAR technology. Considering their mechanisms of action, combined therapy with two or more RC populations when needed, may represent a promising strategy to maintain tolerance. For example, synergistic effects were reported when MSC were used in conjunction with Treg (120). Although RC therapy has the potential to be successful as monotherapy, it may be combined with other existing complementary approaches (anticoagulants, anti-inflammatory, chemical immunosuppression) when appropriate.

Although ACT with RC therapy was successfully evaluated in different clinical transplant settings and offers the possibility for individual therapy with obvious advantages, it is currently not used to induce tolerance to xenotransplanted pig hearts in humans. Additionally, this approach has not yet been assessed in NHP models. This current gap is surprising and will, hopefully, be addressed soon. After overcoming the existing challenges, its large use may become routine to attain tolerance without the need for chronic IS.

Determination of which cell subpopulation should be used for therapy requires a granular understanding of the immune mechanisms (304) at the local heart graft microenvironment. For this, a systemic approach may be necessary to complete an interactive dynamic immune network. This should include interactions at the cellular (immune, stromal, endothelial), humoral (cytokines, antibody), and molecular (metabolic, gene regulatory, protein–protein interaction, signaling) levels, integrated into multiple hierarchical mechanisms. This will hypothetically improve the statistical significance of predictions about the impact of each cellular intervention on graft tolerance induction. Additionally, assessment of combined responses to multiple factors, rather than one stimulus one response approach, will determine the best cellular intervention(s) for maintaining immune graft tolerance. This complexity, in addition to the simultaneous existence of a myriad of intracellular molecular reactions that have been revealed by exhaustive “omics” techniques at the single-cell level (305), suggests the use of artificial intelligence to enhance diagnosis and prognosis of rejection (306) or the development of effective management strategies toward tolerance.

In conclusion, the importance and urgent need to use pig hearts to save human life requires innovative solutions to address chronic xenograft rejection. Specific enhancement of the immunosuppressive mechanisms at the graft level holds the promise of suppressing local immune effector rejection mechanisms without the nonspecific effects of chronic IS treatments. Based on mechanisms of action and advantages, RC therapy is proposed to be one of the best approaches to maintain pig heart xenograft tolerance in humans. More research is required to solve current challenges before their application. Recommended by multianalyte biomarkers, the use of ACT therapy with a specific RC population or off-the-shelf RC product (alone or in combination, once or repeated) has the potential to become a routine personalized treatment to achieve tolerance to xenotransplanted pig heart in the clinical settings. In the future, exosomes or only RC-derived active molecules (or their antagonists) may supplement or replace whole-cell RC therapy.
